# Supplementary figures and images for: Microbes are potential key players in the evolution of life histories and aging in Caenorhabditis elegans
Source: Ecol Evol. 2023 Sep 25;13(9):e10537. doi: 10.1002/ece3.10537 (PMC10518755; doi:10.1002/ece3.10537)

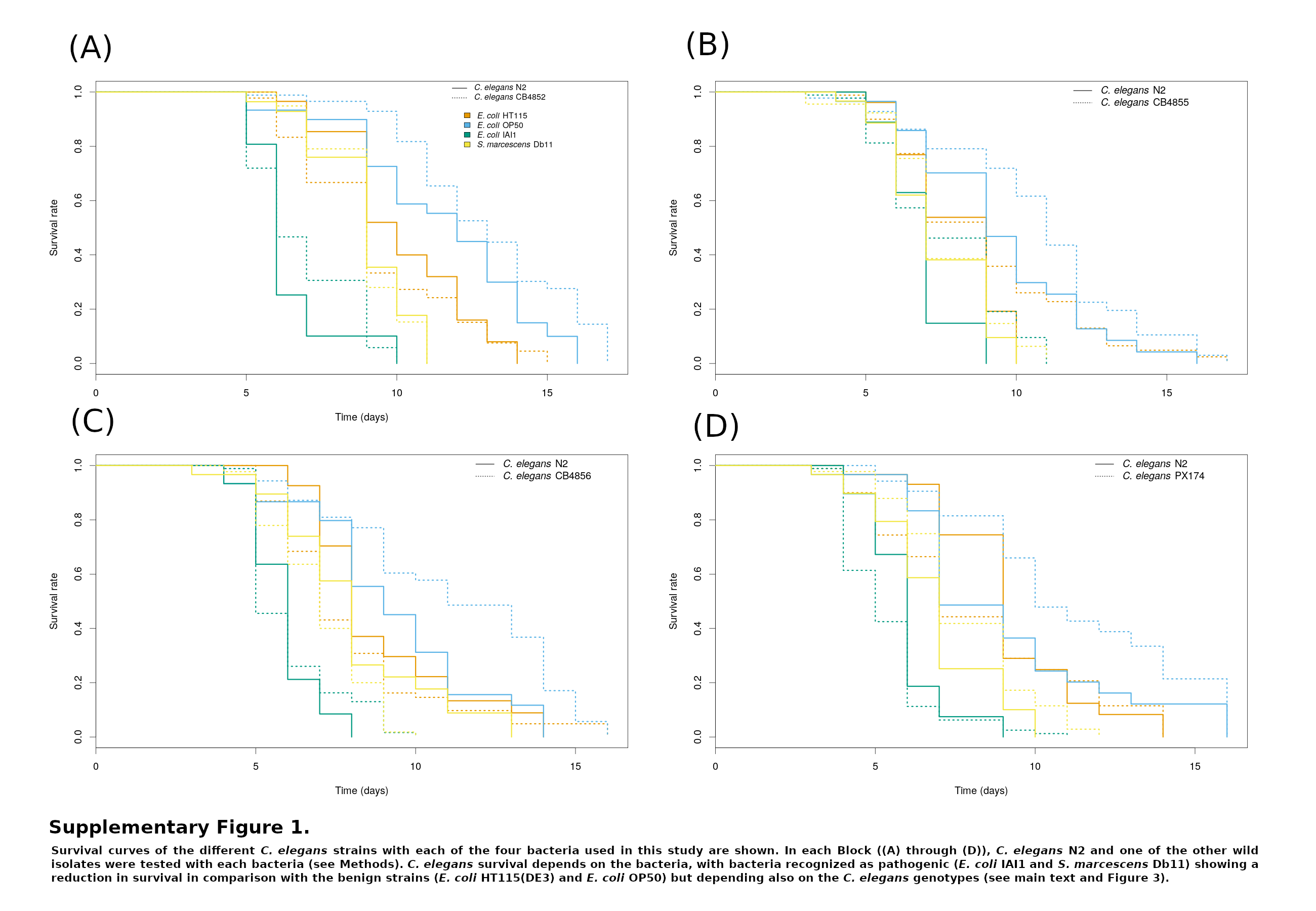

Supplement: Supplementary file 1 — Figure S1. Survival curves of the different C. elegans strains with each of the four bacteria used in this study are shown. [file ECE3-13-e10537-s005.png]

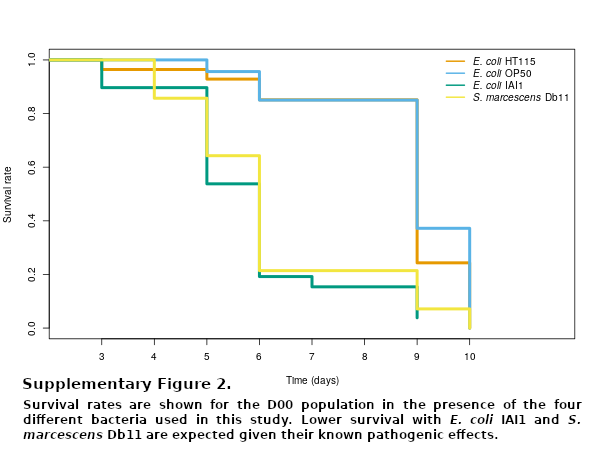

Supplement: Supplementary file 2 — Figure S2. Survival rates are shown for the D00 population in the presence of the four different bacteria used in this study. [file ECE3-13-e10537-s008.png]

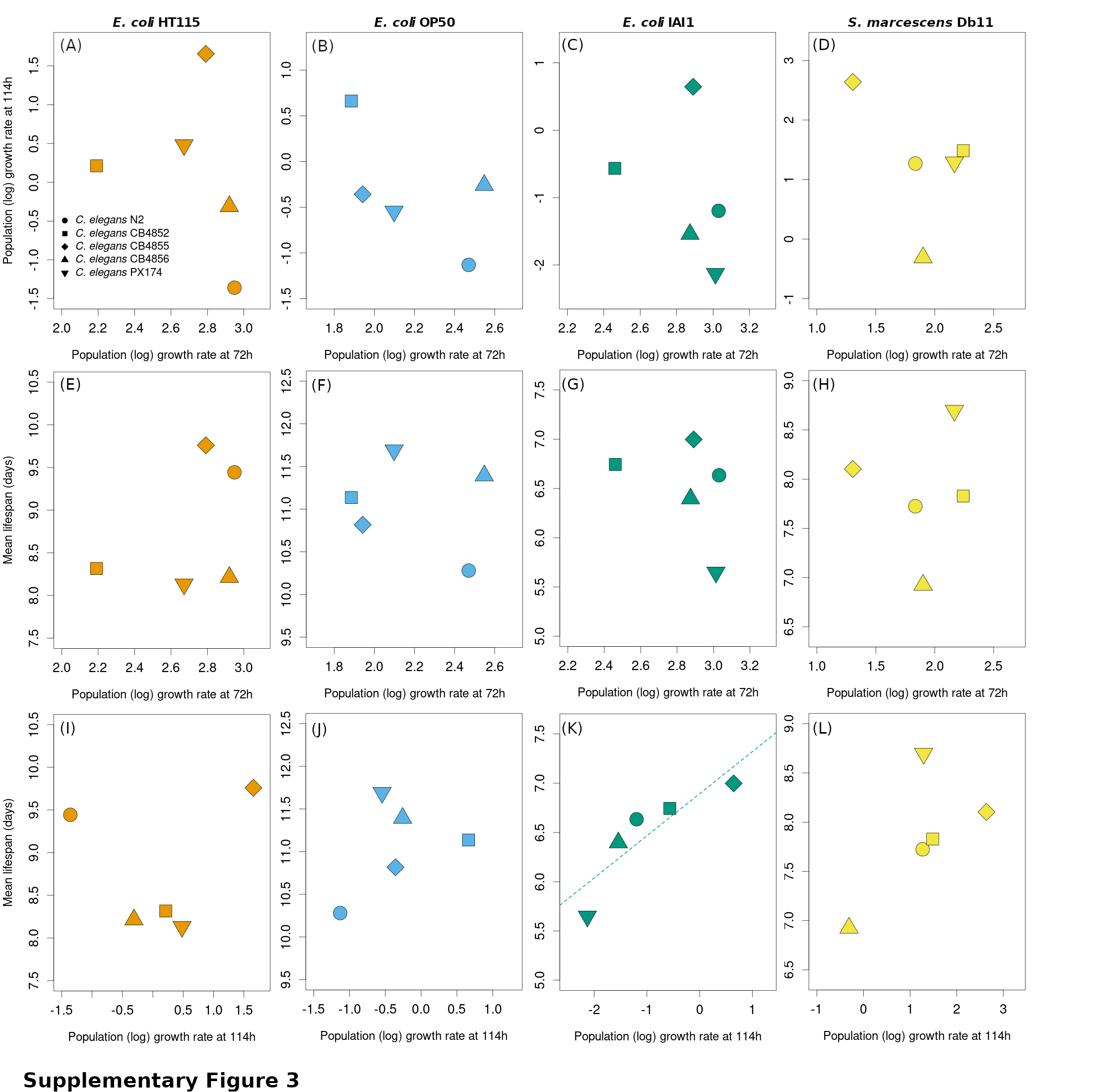

Supplement: Supplementary file 3 — Figure S3. Scatterplots showing the pairwise associations between population growth at 72 h, population growth at 114 h, and mean lifespan obtained for the 5 individual genotypes are shown. In the case where a significant correlation was found (K ‐ population growth at 114 h and lifespan, with E. coli IAI1), the corresponding regression line is shown. [file ECE3-13-e10537-s007.png]
